# Flexibility in Existential Beliefs and Worldview: Testing Measurement Invariance and Factorial Structure of the Existential Quest Scale in an Italian Sample of Adults

**DOI:** 10.3389/fpsyg.2019.02134

**Published:** 2019-09-24

**Authors:** Marco Rizzo, Silvia Testa, Silvia Gattino, Anna Miglietta

**Affiliations:** ^1^Department of Psychology, University of Turin, Turin, Italy; ^2^Department of Human and Social Sciences, University of Aosta Valley, Aosta, Italy

**Keywords:** Existential Quest Scale, existential beliefs, psychometric properties, factorial structure, measurement invariance

## Abstract

The aim of the present study was to assess the psychometric properties of the Existential Quest (EQ) Scale, a nine-items instrument developed to assess openness to changing one’s own convictions concerning existential issues. We developed the Italian version of the scale and examined factorial structure, internal consistency, discriminant validity, and measurement invariance across gender and age groups. A total of 291 Italian adults were recruited, and they completed a self-report questionnaire comprising measures of authoritarianism, cognitive closure, well-being, and religiousness, alongside the EQ. Confirmatory factor analysis showed that the original one-factor structure was replicated in this study, except for one-item that was removed from the subsequent analyses. Both the internal consistency of the eight-item scale as assessed by Cronbach’s α and discriminant validity were in line with those of the original study. However, McDonald’s reliability coefficient were quite low, and further researches employing repeated measures are needed in order to comprehend the contribution of the random error and that of the item specificity in lowering McDonald’s coefficient. Finally, evidence of full measurement invariance across gender and partial measurement invariance across age was obtained. Overall, these findings suggest that the Italian version of the EQ is a promising tool for assessing flexibility about existential issues.

## Introduction

Addressing the fundamental questions of existence – such as the origin and finality of the world, the meaning of life and death, or the existence of transcendence – is a universal human experience that crosses cultures, historical periods, religions, and ideologies, and may be important for optimal individual functioning (Allan and Shearer, 2012; Sullivan, 2013). Indeed, the exploration of existential issues represents a valuable dimension in the promotion of psychological well-being, which reflects the realization of true self, positive relationships, human strengths, and virtues (Ryan and Deci, 2001; Ryff, 2014).

The conceptualization of existential issues is usually relevant to the framework of religion and spirituality (Park, 2005; Zinnbauer and Pargament, 2005). When people consider their global meanings about life and death, they often refer to the sacred aspect that is involved in both the definition of religiousness and spirituality (Pargament et al., 2005; Zinnbauer and Pargament, 2005). The sacred includes concepts such as the divine, God, and the transcendent dimension, which provide an ultimate meaning to life and a sense of personal security and safety toward the unknown (Pargament et al., 2005).

The association between sacred and existential issues might be obvious for religious and spiritual people, but it could be less clear to those who do not attribute great importance to these topics in their lives (Pedersen et al., 2018). Thus, issues related to the global meaning of life should be considered in a broad secular way and not merely centered on a transcendent reality. Indeed, in light of a religious decline in Western societies (la Cour and Hvidt, 2010; Yu et al., 2017), beliefs in science or political ideology could play a role similar to that of religious beliefs for secular individuals (Farias et al., 2013).

Indeed, individual orientations toward a religious, spiritual, or secular perspective (or their possible overlap) do not take place in a social vacuum but rather depend on the cultural context in which a person lives (la Cour and Hvidt, 2010). For example, it has been shown that people living within a collectivist society tend to pursue a religious orientation in the existential experience by conforming to their own religious group, while people in the individualistic society tend to pursue a more secular orientation in the existential experience as a form of navigating personal uncertainty (Sullivan, 2013). In addition, the same individual could think about the global meanings in life in a religious, spiritual, and secular way, depending on his/her different phases of life (la Cour and Hvidt, 2010).

Several studies have attempted to develop measures concerning individual relationships with existential beliefs. For example, Thorne (1973) operationalized the person’s existential status, which included concepts such as existential morale, existential vacuum, existence and destiny, and self-realization. Other scholars have assessed the degree to which people attribute meaning to and are aware of their own lives (Steger et al., 2006; Schulenberg et al., 2011; Richmond, 2015) or have measured individual factors related to existence, such as social and emotional loneliness, existential anxiety, death anxiety, and self-consciousness (Templer, 1970; Scheier and Carver, 1985; DiTommaso et al., 2004; Weems et al., 2004).

However, none of these measures directly assesses the degree to which people could be open to questioning themselves about existential issues, as the Existential Quest (EQ) scale (Van Pachterbeke et al., 2012) does. Perhaps the closest instruments are the Scale for Existential Thinking (Allan and Shearer, 2012) and the Religious Quest Scale (Batson and Schoenrade, 1991a, b). The former, like the EQ, investigates existential issues in a broad sense by assessing the frequency to which people think about these issues. The latter measures flexibility on existential issues but refers only to religious beliefs, and it was created to assess how people redefine their way of being religious as a consequence of contradictions and tragedies in life (Batson and Schoenrade, 1991a, b). Van Pachterbeke et al. (2012) developed the EQ to make a tool that assesses flexibility on existential issues available to all people, regardless of their being religious. To reach this goal, these authors introduced a new broad social-cognitive construct dealing with individual differences in their flexibility to change beliefs on core and universal issues, such as the ultimate meaning of life and the existence of transcendence. This form of open-mindedness could have positive implication at the societal level, because it is related to prosocial attitudes such as tolerance, altruism, and empathy. However, it can also have unfavorable implications at the individual level, because it could be related to feelings of uncertainty and anxiety, as for the religion quest attitude (Van Pachterbeke et al., 2012).

The EQ contains nine items assessing three different components, namely: a relative uncertainty regarding fundamental issues, a valuation of the doubt and questions surrounding these issues, and, eventually, openness to change (or the acknowledgment that one may change his or her own positions and attitudes across time).

In the original work, the authors assessed the factorial structure of the EQ scale and its discriminant validity by means of five studies involving several samples of students from Belgium and Germany and a sample of Belgian adults. As expected by Van Pachterbeke et al. (2012), EQ scores exhibited negative correlations with measures of closed-mindedness and positive correlations with measures related to prosocial attitudes and emotions. In particular, they found a negative correlation with the scores on the need for cognitive closure and Right-Wing Authoritarianism, and positive correlations with a measure of empathy and altruism. Religiousness was weakly correlated or uncorrelated with EQ scores across the studies, according to the hypothesis of independence of the EQ scores from religiousness. Furthermore, as they expected, a negative relationship of EQ scores with age was found (albeit weak). Lastly, as far as gender differences are concerned, no priory expectations were formulated and only in the sample of adults women scored higher than men. The dimensionality of the scale was evaluated by means of explorative factor analysis performed on one of the five studies and then replicated on the whole set of data from the five studies. In the single study, the authors found three factors that isolated religious items, doubt items, and the remaining items, respectively. Whereas on the pooled data a dominant factor of flexibility and a secondary factor dealing with flexibility in worldviews emerged. Supplementary analyses showing that the two factors provided the same pattern of associations with the majority of the variables included in the studies let the authors conclude that the scale could be conceived as unidimensional and that flexibility in worldviews and valuing doubt were facets of the same construct. The internal consistency of the nine items was acceptable (α = 0.74).

The EQ has been applied in different fields of research. For example, Deak and Saroglou (2015, 2017) showed a positive correlation between EQ scores and measures of high tolerance toward moral questions, such as abortion, child euthanasia, gay adoption, and suicide. Furthermore, a negative correlation has been found with a measure of religious fundamentalism (Tapia Valladares et al., 2013), and a positive correlation has been shown with a measure of psychological well-being (Joshanloo, 2017). Finally, Sullivan (2013) showed that people belonging to an individualistic culture obtain higher scores on the EQ than those belonging to a collectivistic culture.

Given the relevance of the issues related to the EQ and, at the same time, the scarcity of instruments that investigate this quest, we consider it useful to deepen the psychometric characteristics of the EQ scale with an Italian sample.

## Aims

The aims of the study were threefold: (1) to examine the factor structure of the Italian adaptation of the EQ; (2) to test the measurement invariance separately across gender and age group; and (3) to assess the discriminant validity of the EQ scores with respect to measures of Right-Wing Authoritarianism (RWA) and the need for cognitive closure. Following Joshanloo (2017), we also tested the relation between EQ scores and a measure of psychological well-being. Furthermore, the relationship with gender, age, and religiousness was considered. To the best of our knowledge, this is the first attempt to confirm the psychometric properties of the EQ scale.

## Materials and Methods

### Participants and Procedure

The participants were 291 Italian adults (64.3% female) aged 19 and 82 years (*M* = 37.0; *SD* = 14.6). Data collection occurred between April 2018 to June 2018; participants were recruited in the Northern part of Italy via a convenience sampling method through the dissemination of the questionnaire among university students attending degree courses in the field of social science (each student delivered some questionnaires to parents and/or acquaintances) (Table 1). The Ethic Committee of the University of Turin approved the study protocol. Participants took part voluntarily after giving their verbal consent to participate in the study. Respondents had to be at least 18 years of age to fill out the questionnaire.

**TABLE 1:** Characteristics as a percentage of the sample.

**Characteristic**	***n* = 291**
**Gender**	
Female	64.3
Male	35.7
**Employment status**	
Students	27.1
Employed	62.2
Unemployed/retired	10.7
**Education level completed**	
Elementary school	1.0
Junior high school	6.2
High school	41.9
Bachelor’s degree or higher	50.9

Data were collected by means of a self-report pencil-and-paper questionnaire that took approximately 20 min to complete. A total of 98.9% of the respondents completed the questionnaire.

### Measures

#### Existential Quest Scale (Van Pachterbeke et al., 2012)

The EQ was translated from English into Italian collegially by the authors and then was back translated by a native speaker. Participants were required to respond on a 7-point scale ranging from 1 (strongly disagree) to 7 (strongly agree). In the current study, Cronbach’s alpha was 0.68. The original English items and the Italian adaptation of the EQ are reported in Appendix 1.

#### Right-Wing Authoritarianism Scale (Funke, 2005; Roccato et al., 2009)

The RWA is a 12-item self-report scale that assesses an overall authoritarianism attitude, rated on a 5-point scale ranging from 1 (strongly disagree) to 5 (strongly agree). Cronbach’s alpha found in the current study was 0.75.

#### Need for Cognitive Closure Scale-Brief Form (Pierro et al., 1995; Roets and Van Hiel, 2011)

We used a brief form (15 items) of the original scale of Webster and Kruglanski (1994), which assesses overall individual differences in cognitive closure. Participants responded on a 6-point scale ranging from 1 (not at all characteristic of me) to 6 (entirely characteristic of me). Cronbach’s alpha found in the current study was 0.84.

#### The Mental Health Continuum-Short Form (Keyes, 2002; Petrillo et al., 2015)

The Mental Health Continuum-Short Form (MHC-SF) assesses three major dimensions of well-being: *psychological*, *social*, and *emotional*. Participants were asked to indicate how much of the time during the last month they functioned in a specific manner. Items were rated on a 6-point scale ranging from 0 (never) to 5 (always). The internal consistency found in the current study was good, Cronbach’s alphas ranged from 0.77 to 0.82.

#### Religiousness

By means of principal component analysis, we calculated an index through three items created for the purpose of this study: “How much important is religion for you?,” “Apart from weddings and funerals, how often do you attend mass or, if not Catholic, other religious rituals?,” “How often do you attend the activities/initiatives of your religious group?.” The items were rated on a 6-point scale ranging from 0 (not at all/never) to 5 (very much/more than once a week). In the current study, Cronbach’s alpha was 0.84.

A brief list of sociodemographic items, including respondents’ gender, age, and education, was also included.

We developed two versions of the questionnaire, presenting the EQ before and after the RWA and Need for Cognitive Closure Scale-Brief form (NFCS-BF) to prevent potential order effects. The MHC-SF was the first scale in both questionnaires.

### Statistical Analyses

Imputation was performed using the expectation maximization (EM) method after it was verified that the missing values of the scales, ranging from 1.3 to 3.1%, were missing completely at random (MCAR) (Little, 1998).

We performed confirmatory factor analyses using MPLUS 7.3 (Muthén and Muthén, 1998-2015) to assess the factorial structure of the scale. According to the original study, we estimated a unidimensional model.

Because the data violated the multinormality condition [Mardia’s multivariate omnibus test of skewness and kurtosis (2.26) = 125.60, *p* < 0.001], we used the Asparouhov and Muthén (2010) mean- and variance-adjusted ML (MLMV). As found by Maydeu-Olivares (2017), this estimation method has good properties in terms of the accuracy of standard errors and type I error in the presence of non-normal data. The following criteria were used to evaluate the acceptability of the goodness of fit of the model: root mean square error of approximation (RMSEA) ≤ 0.08; comparative fit index (CFI) ≥ 0.90; standardized root mean square residual (SRMR) ≤ 0.08 (Browne and Cudeck, 1993; Hu and Bentler, 1999). To assess measurement invariance, a multiple-group CFA (with gender and age as the grouping variables) was performed, and four increasingly restrictive models were estimated (Vandenberg and Lance, 2000). In the first model, all parameters were freely estimated across groups (configural invariance); in the second model, the loadings were assumed to be equal across groups (metric invariance); in the third model, both loadings and intercepts were constrained to be equal across groups (scalar invariance); and finally, in the fourth model, the residual variances were assumed to be equal across groups. The goodness of fit of each model was compared to that of the previous model (e.g., 2° vs. 1°; 3° vs. 2°). According to Chen (2007), the following changes in goodness-of-fit indices were considered indicative of a lack of invariance: ΔCFI ≤ −0.005; ΔRMSEA ≥ 0.010; regarding the SRMR, the cut-off was 0.025 for loading invariance and 0.005 for intercepts and uniqueness invariance.

The discriminant validity of the scale scores was tested by means of correlations (Pearson’s *r*). Scale reliability was evaluated by means of the traditional Cronbach’s α and by the Omega coefficient (ω, McDonald, 1978). As it is well known, α furnishes an unbiased estimate of reliability only when items conform to the essential tau-equivalence model under the Classical Test Theory (i.e., when items scores fit a unidimensional model in which the loadings are set to be equal and errors are uncorrelated). An appropriate alternative to α is the Omega coefficient (McDonald, 1999) that is based on the unidimensional model estimates and it is defined as the ratio between the variance due to the common factor and the variance of the total scale scores. In particular, the coefficient for measures with correlated errors was computed (Raykov and Marcoulides, 2016, p. 304).

All the analysis, except for CFAs, were performed with SPSS 25.0 (IBM SPSS Statistics, IBM Corporation).

## Results

### Confirmatory Factor Analysis

The estimation of the one-factor model produced an unsatisfactory fit to the data: χ^2^(27) = 150.1, *p* < 0.01; RMSEA = 0.125 (90% CI = 0.11, 0.14); CFI = 0.639; and SRMR = 0.085.

To improve the model fit, we considered the contents of the items, looking for pairs of items that eventually shared part of their specificity. This examination identified three pairs of items that were more similar to each other than to the other elements of the scale. In detail, the pairings of items were as follows: items 1 and 7, the only items addressing the goal of life; items 2 and 9, the sole items related to the religious and spiritual sphere; and items 3 and 4, the unique items concerning the valorization of doubt. On the grounds of this consideration, with the support of the modification indices (MIs), the model was retested after the residuals of each item pair were correlated (1–7; 2–9; 3–4). The result of this model was satisfactory in terms of global fit indices: χ^2^(24) = 51.5, *p* < 0.01; RMSEA = 0.063 (90% CI = 0.04, 0.09); CFI = 0.919; and SRMR = 0.046.

As shown in Table 2, factor loadings (standardized values) were acceptable (>0.30), except for items 9 and 7, and all estimates were statistically significant (*p* < 0.05). The correlations between residuals were also not negligible (>0.30).

**TABLE 2:** Standardized loadings for one-factor confirmatory model of Existential Quest Scale (*n* = 291).

**Item**	**Loading**
1. Today, I still wonder about the meaning and goal of my life	0.43
2. My attitude toward religion/spirituality is likely to change according to my life experiences	0.39
3. Being able to doubt about one’s convictions and to reappraise them is a good quality	0.45
4. In my opinion, doubt is important in existential questions	0.50
5. My way of seeing the world is certainly going to change again	0.71
6. My opinion varies on a lot of subjects	0.52
7. I know perfectly well what the goal of my life is^∗^	0.16
8. Years go by, but my way of seeing the world doesn’t change^∗^	0.41
9. I often reappraise my opinion on religious/spiritual beliefs	0.24

*^∗^Item reverse-coded. Model estimates include three correlations between residuals: 0.48 (items 2 and 9); 0.38 (items 3 and 4); and 0.30 (items 1 and 7). All estimates are statistically significant at *p* < 0.05.*

### Measurement Invariance

The unidimensional model with three residual covariances obtained in the previous analysis was estimated in the multiple-group CFA to evaluate the degree of measurement invariance of EQ items across gender and age group.

The model imposing configural invariance across gender showed satisfactory fit values: χ^2^(48) = 67.4, *p* < 0.05; RMSEA = 0.053 (90% CI = 0.01, 0.08); CFI = 0.939; and SRMR = 0.055. However, a close examination of the loadings showed that, in the group of men, the loading of item 7 was not statistically significant (0.05; *p* = 0.84). Thus, we excluded item 7 from the analysis of gender invariance, and this exclusion reduced the number of residual covariances to be estimated: the covariance between items 1 and 7 was no longer a model parameter. As shown in Table 3, on the remaining eight items, all the models – from the one that imposes equality of the loading pattern (configural) to the one that imposes equality of all item parameters (uniqueness invariance) – showed excellent fit to the data. The non-significant difference in χ^2^ (Δχ^2^) and the very small change in RMSEA, CFI, and SRMR obtained in each of the comparisons lend support to the idea that the EQ items exhibit full measurement invariance across gender.

**TABLE 3:** Measurement invariance of the EQ scale.

**Models across gender**	**χ^2^**	***df***	**RMSEA**	**CFI**	**SRMR**	**Δχ^2^**	**Δ*df***	**ΔRMSEA**	**ΔCFI**	**ΔSRMR**
Males	25.3	18	0.062	0.928	0.060	–	–	–	–	–
Females	20.3	18	0.026	0.988	0.036	–	–	–	–	–
1. Configural	45.6	36	0.043	0.967	0.046	–	–	–	–	–
2. Metric_a_	53.9	45	0.037	0.969	0.051	8.73	9	–0.006	0.002	0.005
3. Scalar_a_	60.0	52	0.032	0.972	0.055	5.76	7	–0.005	0.003	0.004
4. Uniquenesses_a_	68.8	60	0.032	0.970	0.059	9.50	8	0.000	–0.002	0.004
**Models across age**										
Adults (aged ≥ 31 years)	20.4	18	0.030	0.984	0.043	–	–	–	–	–
Young adults (aged < 31 years)	28.8	18	0.065	0.923	0.049	–	–	–	–	–
1. Configural	48.5	36	0.049	0.957	0.046	–	–	–	–	–
2. Metric_a_	59.5	45	0.047	0.951	0.058	11.74	9	–0.002	–0.006	0.012
3. Scalar_a_	75.4	52	0.056	0.920	0.070	18.11^∗^	7	0.009	–0.031	0.012
3a. Scalar_a,b_	65.3	50	0.046	0.948	0.062	5.92	5	–0.001	–0.003	0.004
4. Uniquenesses_a_	79.8	58	0.051	0.926	0.077	15.64^∗^	8	0.005	–0.022	0.015
4a. Uniquenesses_a,c_	70.5	56	0.042	0.951	0.066	6.11	6	–0.004	0.003	0.004

*RMSEA, root mean square error of approximation; CFI, comparative fit index; SRMR, standardized root mean square residual. ^*a*^The error covariance between items 2 and 9 and between items 3 and 4 was constrained to be equal across groups; ^*b*^Free intercept on items 8 and 1; ^*c*^Free uniqueness on items 8 and 1. ^∗^*p* < 0.05.*

With the aim of assessing measurement invariance with respect to age, two groups were formed using the median of the sample (31 years) as a cut-off (young adults, *N* = 142; adults, *N* = 149). The fit of the configural model on the nine items of the scale was adequate [χ^2^(48) = 66.8, *p* < 0.05; RMSEA = 0.052 (90% CI = 0.01, 0.08); CFI = 0.939; SRMR = 0.053]. However, as in the gender group analyses, the loading of item 7 was not statistically significant; in this case, it was not statistically significant in either of the two groups (young adults: 0.28, *p* = 0.14; adults: 0.07, *p* = 0.74). Thus, we also dropped item 7 in this analysis. As shown in Table 3, the configural and metric models provided excellent fit to the data. In terms of changes in the fit measures, in the metric invariance model, only ΔCFI was slightly above the cut-off, but we did not consider this lack of fit to be problematic because all the other changes in fit indices were small. The imposition of the equality of the intercepts resulted in a remarkable change in both the CFI and SRMR. To evaluate whether partial scalar invariance was tenable, we examined the MIs relative to the item intercepts, and we relaxed the equality constraint on the item intercept associated with the largest MI, one at a time, until the changes in the fit indices with respect to the metric invariance model were negligible. After the intercept equality constraint on items 8 and 1 was removed, changes in the fit indices were very small. Regarding the uniqueness invariance, both ΔCFI and ΔSRMR were outside the range. The inspection of MI suggested the removal of the equality constraint from the uniqueness of items 8 and 1, thus leading to a satisfactory model fit. These two items were not invariant across age groups, and both items exhibited lower intercept and greater uniqueness in the adult sample than in the younger sample.

### Discriminant Validity

To correlate EQ scores with those of the other scales, a total mean score of flexibility was computed. In light of the results obtained above, item 7 was excluded from the computation (means and standard deviations of EQ items are shown in Appendix 2).

As reported in Table 4, EQ scores showed a moderate negative correlation with RWA scores and a weak negative correlation with NFCS-BF scores. Flexibility scores were not correlated with well-being scores, neither with subscales nor with total scores.

**TABLE 4:** Summary of intercorrelations for scores on the EQ and the other study variables.

	**1**	**2**	**3**	**4**	**5**	**6**	**7**	**8**	**9**	**10**
1. EQ	–									
2. RWA	–0.38^∗∗^	–								
3. NFCS	−0.14^∗^	0.39^∗∗^	–							
4. MHC	–0.06	0.07	–0.15^∗∗^	–						
5. EWB	–0.04	–0.01	–0.17^∗∗^	0.79^∗∗^	–					
6. SWB	–0.01	0.05	–0.16^∗∗^	0.86^∗∗^	0.53^∗∗^	–				
7. PWB	–0.09	0.10	–0.09	0.91^∗∗^	0.65^∗∗^	0.63^∗∗^	–			
8. Religiousness	–0.08	0.36^∗∗^	–0.02	0.27^∗∗^	0.15^∗∗^	0.32^∗∗^	0.21^∗∗^	–		
9. Gender (0 = M)	0.07	–0.04	0.01	–0.01	0.03	–0.03	0.02	0.02	–	
10. Age	–0.21^∗∗^	0.24^∗∗^	0.24^∗∗^	–0.01	0.05	–0.11	0.09	0.01	–0.07	–

*EQ, Existential Quest Scale without item 7; RWA, Right-wing Authoritarianism; NFCS, Need for Cognitive Closure Scale-Brief form; MHC, Mental Health Continuum-Short form (total score); PWB, Psychological well-being; SWB, social well-being; EWB, emotional well-being. *^∗^p* < 0.05; ^∗∗^*p* < 0.01.*

Regarding the religiousness index, no correlation was found, and no relationship emerged with respect to gender. Flexibility scores were negatively correlated with age, although the correlation was weak.

### Internal Consistency

For the 8-items scale, Cronbach’s α was 0.70 and McDonald’s ω was 0.61, meaning that 61% of the total score variance was due to the common latent factor. The difference between α and ω was mainly due to the presence of correlated errors. In fact, when omega was computed including the error covariances among the systematic part at the numerator of the formula:

$$\frac{{\left(\sum \lambda_{i}\right)}^{2}+2^{*}\,\sum \sigma_{i,j}}{{\left(\sum \lambda_{i}\right)}^{2}+2^{*}\,\sum \sigma_{i,j}+\sum \sigma_{i}^{2}},$$

the value (0.69) was very close to that of α.

## Discussion

The study investigated the psychometric properties of the EQ across an Italian sample. The results supported the unidimensionality of the scale, in line with the findings of the original study of Van Pachterbeke et al. (2012). More specifically, scale scores were essentially unidimensional, because the presence of some error covariances signals that there are some secondary dimensions. However, this result is consistent with the intention of the proposers of the scale to develop a broad measure of flexibility by using a set of items “that do not merely paraphrase each other, including items that address the different components of the quest orientation” (Van Pachterbeke et al., 2012, p. 3). The presence of more than one item for each component created undesired covariation between items (as for the two items about religious beliefs and the two relative to evaluating doubt). At the same time, the number of items per component was too small to substantiate the presence of a general factor and some content-related factors (group factors).

One item (“I know perfectly well what the goal of my life is”) performed poorly both in the factor analysis conducted on the whole sample and in the measurement invariance tests across gender and age groups. This result was in line with those of previous studies that found this item to be a poor indicator of existential flexibility (Van Pachterbeke et al., 2012; Joshanloo, 2017). In light of these considerations, we do not advise the consideration of item 7 in the EQ.

The 8-item scale revealed full measurement invariance across gender, reflecting that there are no differences in the Italian sample between males and females in the EQ factorial structure, while partial invariance emerged across age groups because two items (items 1 and 8) differed both in terms of intercept and residual variance across younger and older adults. Considering the contents and formulations of these items, some considerations can be formulated. It is plausible that being uncertain about the meaning of life (item 1) has different implications for younger and older adults. For younger people more than for older people, it could be a positive aspect associated with the openness to new experiences, whereas for older than for younger people, it could have a negative, depressive connotation. Similarly, the significance of the item about changing the way of seeing the world (item 8) may have a different meaning according to the age of respondents, especially because this item refers to change occurring “over the years”.

In line with the original study (Van Pachterbeke et al., 2012), EQ scores showed good discriminant validity in terms of their correlation with RWA and NFCS-BF scores. High flexibility in EQ was associated with the tendency to be autonomous with respect to norms (low RWA) and to be less cognitively rigid (low need for cognitive closure). Furthermore, consistent with the literature, we found that younger people are more flexible with respect to existential questions than older people are.

As concern internal consistency of the total scale score, α-value was similar to that obtained in the original study (α = 0.74), and quite higher than the value of omega. Thus, our results are coherent with those of Gu et al. (2013) who found that α tends to treat correlated error variance as true variance and thus inflates the estimate of reliability. The value of omega was low, but this result does not imply necessarily that the scale is heavily affected by random error variation. The low value could be mainly due to item specificity that is, the influence of factors that are specific for each item. The item specificity is a source of systematic variation that could be considered a component of the “true” variance, depending on the definition of reliability the researcher is adopting. Even if in a single administration, as in the present study, it is not possible to distinguish between random variation and items specificity, we can conjecture that specificity is a not negligible component of EQ scores because, as stated above, EQ was intended as a broad measure of flexibility.

In summary, the present study assessed for the first time the factorial structure of the EQ by means of a confirmatory approach. The study provided some evidence of measurement invariance across gender and age and showed that the Italian version of the scale presents satisfactory psychometric properties. Nonetheless, this study is not exempt from some limitations. Firstly, because of the type of sampling method employed, the participants were not representative of the Italian population, with an over-representation of women and high educated people. Secondly, although the number of participants was adequate to perform the intended analyses, it did not allow for the formation of more than two age groups, thus limiting the exploration of the functioning of the items according to age. Moreover, it did not allow splitting the sample and performing both exploratory and confirmatory factor analyses. The exploratory approach with a bi-factor rotation could be useful in further exploration of the factorial structure of the scale, because it allows modeling a general factor and two or more group factors related to the content components of the scale. We could not estimate a confirmatory bi-factor model because a minimum of three indicators for each group factor is request. Moreover, further researches aimed at assessing EQ reliability by means of a test–retest design are recommendable in order to assess how much EQ total score is affected by random variation (McCrae, 2015). Finally, although promising, we have collected data in a predominantly Catholic country, so it is necessary to investigate the properties of this scale in other countries with different cultural and religious traditions.

## Conclusion

The new construct and the relative scale developed by Van Pachterbeke et al. (2012) could be used in several field of psychology (social, clinical, developmental) as it deals with issues that more or less involve all human beings in every period of life since the development of abstract and critical thinking.

The EQ scale may represent a useful tool to better understand how people experience different perspectives in Western societies characterized by the coexistence of different cultures and religions. Assessing individual differences in their flexibility on existential issues could help to understand why some people are willing to accept the presence of people with different cultures and/or religions and others tend to do not tolerate the contradiction due to the multicultural presence.

At the individual level, being more or less an existential quester could be related to personal well-being. In contrast to previous studies that reported a positive correlation between the two measures (Joshanloo, 2017), our results failed to find a significant relationship between the EQ and individuals’ well-being. Indeed, high flexibility with respect to the EQ could combine with emotional instability and anxiety, as claimed in the original study (Van Pachterbeke et al., 2012). In other words, future studies could aim to disambiguate the positive or negative contribution of such flexibility in individuals’ lives, as flexibility may help manage stressful situations such as disabling illness (la Cour, 2008) but could also be related to existential anxiety and an increase in risky behaviors during adolescence (Carter et al., 2013).

## Data Availability

The datasets generated for this study are available on request to the corresponding author.

## Ethics Statement

The studies involving human participants were reviewed and approved by the Ethic Committee of the University of Turin (code 10039). The patients/participants provided their written informed consent to participate in this study.

## Author Contributions

MR, ST, SG, and AM conceived the study. MR and ST did the analyses. MR wrote the manuscript. All authors discussed the results together and contributed to the final manuscript, doing critical revisions and giving suggestions, and approved the submitted version of the manuscript.

## Conflict of Interest Statement

The authors declare that the research was conducted in the absence of any commercial or financial relationships that could be construed as a potential conflict of interest.

## Appendix 1

### Existential Quest (English Version in Brackets)

1. Ad oggi, mi pongo ancora delle domande sul significato e lo scopo della mia vita [Today, I still wonder about the meaning and goal of my life].
2. Sulla base delle esperienze della mia vita, il mio approccio verso la religione/spiritualità probabilmente cambierà [My attitude toward religion/spirituality is likely to change according to my life experiences].
3. Mettere in dubbio le proprie convinzioni e rivalutarle è una caratteristica positiva [Being able to doubt about one’s convictions and to reappraise them is a good quality].
4. Penso che il dubbio abbia un ruolo importante nelle domande esistenziali [In my opinion, doubt is important in existential questions].
5. Il mio modo di vedere il mondo sicuramente cambierà ancora [My way of seeing the world is certainly going to change again].
6. La mia opinione su molti argomenti varia [My opinion varies on a lot of subjects].
7. Ho ben presente qual è lo scopo della mia vita [I know perfectly well what the goal of my life is](^∗^).
8. Passano gli anni ma il mio modo di vedere il mondo non-cambia [Years go by, but my way of seeing the world doesn’t change](^∗^).
9. Spesso rivaluto la mia opinione sulle credenze religiose/spirituali [I often reappraise my opinion on religious/spiritual beliefs]

(^∗^) reverse-scored item.

## Appendix 2

**TABLE:** Descriptives of the Existential Quest Scale

**Item**	***M***	***SD***	**Skew**	**Kurt**
1	5.21	1.81	–0.90	–0.22
2	3.43	1.93	0.26	–1.09
3	5.95	1.31	–1.20	0.71
4	5.88	1.26	–1.23	1.31
5	5.55	1.34	–1.04	1.11
6	4.78	1.53	–0.24	–0.84
7^∗^	3.85	1.87	0.07	–1.07
8^∗^	5.05	1.71	–0.82	–0.21
9	3.00	1.81	0.61	–0.81
Scale scores	4.74	0.87	–0.23	–1.18
Scale scores^a^	4.85	0.91	–0.31	–0.07

*^∗^Reverse-scored item. ^*a*^Scale scores without item 7.*
